# Molecular genetic association of rs8099917 and rs1800795 polymorphisms in the progression of hepatitis Delta virus liver disease

**DOI:** 10.1590/1678-9199-JVATITD-2023-0025

**Published:** 2024-01-12

**Authors:** Ana Maísa Passos-Silva, Eugênia de Castro e Silva, Lourdes Maria Pinheiro Borzacov, Adrhyan Araújo, Anita Sperandio Porto, Juan Miguel Villalobos Salcedo, Deusilene Vieira

**Affiliations:** 1 Molecular Virology Laboratory, Oswaldo Cruz Foundation Rondonia - FIOCRUZ/RO, Porto Velho, RO, Brazil.; 2 Postgraduate Program in Experimental Biology, Federal University of Rondonia, UNIR/FIOCRUZ-RO, Porto Velho, RO, Brazil.; 3 Federal University of Rondonia, UNIR, Porto Velho, RO, Brazil.; 4 Tropical Medicine Research Center - CEPEM, Porto Velho, RO, Brazil.; 5 National Institute of Epidemiology of the Western Amazon, INCT EpiAmO, Porto Velho, RO, Brazil.; 6 Unir Pathology Laboratory, UNIR/RO, Porto Velho, RO, Brazil.

**Keywords:** Polymorphisms, Chronic liver disease, Hepatitis Delta Virus

## Abstract

**Background:**

The relationship between viral infections and host factors holds high hopes for identifying the role of Interferon Lambda 3 (IFNL3) and Interleukin 6 (IL-6) polymorphisms in the development of Chronic Liver Disease (CLD) in patients infected with hepatitis Delta virus (HDV) in the Western Brazilian Amazon.

**Methods:**

Cross-sectional study conducted with a cohort of 40 chronic HDV patients, 27 with CLD and 13 without evident liver damage. Biological samples from the participants were analyzed using the polymerase chain reaction (PCR) technique, followed by sequencing by the automated Sanger method.

**Results:**

The rs8099917 T allele, from the IFNL3 gene, showed a higher frequency in both groups; however, it was not possible to establish an association with HDV infection [OR = 1.42 (0.42 - 4.75; p = 0.556 (95% CI). For IL-6, the rs1800795 G allele was superior to rs1800795 C. Analyzing both distributions in the studied groups, any association with HDV was absent (p > 0.05).

**Conclusion:**

The results suggest that the rs8099917 T/G (IFNL3) and rs1800795 G/C (IL-6) polymorphisms are not associated with the evolution of HDV in the studied population.

## Background

The pathogenesis of viral infections is determined by a complex relationship between the viral agent and intrinsic host factors [1]. Among the factors that may be associated with clinical evolution, host genetic variation has been studied as a possible determinant in the pathogenicity of viral infections [2].

In humans, the great genetic variation is organized in the form of Single Nucleotide Polymorphisms - SNPs, the result of point mutations, substitutions, or deletions, which produce base-pair differences between chromosome sequences [2]. Some of these SNPs have been associated with remarkable environmental adaptations, such as splenomegaly and increased tolerance to hypoxia in individuals from the Bajau population, also known as sea nomads, whose subsistence depends on food collected by free diving [3].

The possible influence on human health conditions may be related to the location of SNPs, which can be detected in coding and non-coding regions. Depending on the region of variability, interference may occur in the regulation of transcription; structure, stability, and expression of RNAs; protein expression, and in the final confirmation of gene products. Therefore, their clinical importance lies in their possible use as biomarkers and aid in [1] localizing genes associated with various diseases, [2] predicting population responses to drugs or vaccines, and notably, [3] anticipating the response of individuals to a given disease [4, 5]. 

Some SNPs have been described as influencing factors in the clinical course of viral hepatitis B and C. For example, evidence has been presented that links some alleles of SNP rs8099917 located on chromosome 19 in the exon of the Interferon lambda 3 (IFNL3) gene near the non-coding region [6, 7], associated to predisposition of chronic hepatitis B virus (HBV) infection susceptibility; to the level of fibrosis in histological findings in hepatitis C virus (HCV) infection; and the response to treatment with α-PEG-IFN/RBV in hepatitis C [8, 9]. In addition to the description of the influence of this SNP in other pathologies such as COVID-19 where alleles are related to the severity of cases [6]. 

Other SNPs are important in the context of viral hepatitis, such as rs1800795 located near the promoter region of the Interleukin 6 (IL-6) gene on chromosome 7 [10], whose position can harbor alleles and genotypes that, through effects on gene transcription, directly affect the levels of this cytokine [11]. It has been shown that this cytokine seems to contain a potential genetic factor for the development of liver diseases. There are associations of this SNP concerning HCV-mediated liver disease [12], in addition to the relationship of genetic influence on progression in other diseases such as COVID-19 [13]. Elucidating the clinical and virological aspects of Hepatitis Delta, this pathology presents a peculiar infectiousness condition: it arises only when associated with hepatitis B. Briefly, HDV is a defective virus that uses the HBV envelope during particle replication inside hepatocytes to become infectious, and thus becomes dependent on the presence of HBV as an auxiliary virus [14]. When coinfected with HDV, progression to fibrosis is more rapid and takes less than 5 years [15, 16] in cirrhotic patients, the chances of developing HCC are increased threefold, and the mortality risk is doubled compared to a patient with HBV mono infections [17]. 

The World Health Organization (WHO) estimates that there are about 296 million chronic HBV carriers worldwide [18], where approximately 15 million may have serologic evidence for coinfection with hepatitis Delta virus, and HDV is dispersed across the globe, but with its distribution pattern does not uniform [19]. The Amazon region is considered to have high endemicity for HDV [15, 20]. In Brazil, about 73,1% of the recorded cases of hepatitis Delta occurred in the northern region of the country. This area comprises most of the western Brazilian Amazon [21], with circulation of HBV genotypes A, D, F, and HDV genotype 3 in the region [22, 23].

Considering the medical and epidemiological importance of HDV infection in Western Amazon; the remarkable influences of SNPs rs8099917 (IFNL3) and rs1800795 (IL-6) against HBV and HCV infections and liver disease development [5]; lack of studies on their influence in HDV infection, this study aims to evaluate possible influences of these SNPs on the development of chronic liver disease (CLD) associated with Delta hepatitis in patients residing in locations of Western Amazon. Additionally, we evaluated correlations between genetic profiles and demographic aspects of gender, age, and alcoholism with the development of CLD, as well as thrombocytopenia, which, therefore, may influence the clinical progression of coinfection between HBV and HDV.

## Methods

### Ethics declarations

This study was approved by the research ethical committee from Tropical Medicine Research Center - CEPEM/RO (nº 3.826.726), and informed consent was obtained from all participants. 

### Type and location of the study

This is a descriptive, cross-sectional study, using convenience sampling, where patients diagnosed with a chronic profile for Hepatitis Delta Virus (HDV) infection were included. The analyses were performed in the Molecular Virology Laboratory, Oswaldo Cruz Foundation Rondônia - FIOCRUZ/RO in collaboration with the Specialized Ambulatory for Viral Hepatitis (AHV/RO) of the Rondônia Tropical Medicine Research Center (CEPEM/RO), Brazil.

### Population of study

The study population was made up of a sample of 40 individuals who were already being clinically monitored by the Specialized Ambulatory for Viral Hepatitis with positive anti-HDV serology. They were non-indigenous and did not have co-infection with HIV or HCV, stratified into two groups: 27 HDV-infected individuals with a profile of Chronic Liver Disease (CLD) and 13 individuals with HDV infection without a profile of alterations in the liver parenchyma. All participants signed an informed consent form.

Participants underwent medical consultation, followed by a collection of biological samples. Hematological tests were performed (specifically platelet count and determination of prothrombin activity time); biochemical tests (level of liver transaminases, bilirubin, albumin, alkaline phosphatase); imaging (whole abdomen ultrasound and upper digestive endoscopy) and liver biopsy and/or elastography when clinically indicated. 

Individuals who had fibrosis levels equal to or greater than FIB-2 in biopsy results, the presence of esophageal varices in upper digestive endoscopy exams, or the presence of alteration in the liver parenchyma associated with alteration suggestive of portal hypertension in abdominal ultrasound examination were classified as having chronic liver disease - CLD when, in addition to one of the results mentioned above, were associated with results of altered biochemical liver function tests (elevated bilirubin, transaminases and alkaline phosphatase and decreased levels of serum albumin).

### Molecular characterization of single nucleotide polymorphisms (SNPs)

*Extraction of Genomic DNA*

The commercial PureLink®Genomic DNA Mini Kit (Invitrogen) extraction kit was used for gDNA (genomic deoxyribonucleic acid) extraction using 200 μl of whole blood samples according to the manufacturer's instructions. The product was solubilized in a specific buffer and stored at minus 80 ºC.

*Conventional Polymerase Chain Reaction and Sequencing*

For PCR, reference sequences were used in Table 1, consisting of 5 p/moL of the oligonucleotide, where 1 µl of each dNTP (1.5 mM), 0,75 µl of MgCl_2_ (1.5 mM), and 0.75 µL of 5 U/µL of Taq DNA Polymerase were added, using 210 ng/µL of extracted genomic DNA, per reaction.

**Table 1.** Candidate genes and oligonucleotide sequences (primers) for amplification of the fragments of interest in the PCR process.

Gene	Polymorphism	Sequence	References
IFNL3	rs8099917	5’-TTGTCACTGTTCCTCCTTTTGTTT-3’ 5’-TGGGAGAATGCAAATGAGAGATA-3’	[24]
IL-6	rs1800795	5’-AAAGGAAGAGTGGTTCTGCTTCT-3′ 5′- ATCTTTGTTGGAGGGTGAGG-3′	[25]

The conventional PCR product was purified using ExoSAP-IT™ PCR Product Cleanup (Applied Biosystems™, California, USA), according to the manufacturer's instructions, and sequenced using the automated Sanger method, carried out in partnership with the DNA Sequencing Technology Platform (RPT01B IGM) of the Oswaldo Cruz Foundation Bahia - FIOCRUZ/BA. Sequencing result was analyzed in the software MEGA7 - Molecular Evolutionary Genetic Analysis, through direct visualization of the electropherogram to determine the position of the SNPs (rs8099917 and rs1800795). The PhRed score values of the electropherogram, visualized in the online tool Electropherogram Quality Analysis, were also evaluated to increase the reliability of the bases determined by sequencing in the positions of these SNPs, prioritizing sequences whose PhRed values in these positions were equal or greater than 30 (one error probability in 1000). In addition, the bioinformatics tool SNPedia was used to confirm data regarding polymorphisms.

### Statistical analysis

For the statistical analyses, GraphPad Prism^®^ 8.4.0 statistical software was used. Categorical variables were compared by chi-square or Fisher's exact test, and for continuous variables medians, minimum and maximum were used. To assess the allelic and genotypic frequency of polymorphisms, a Chi-square test (χ^2^) was carried out for the Hardy-Weinberg equilibrium hypothesis, considering a significance level of 3.84 with a degree of freedom of one. A Two-way ANOVA test was performed for comparison between groups. The p values < 0.05 were considered statistically significant.

## Results

### Epidemiological and clinical data of the study population

**Figure 1.**
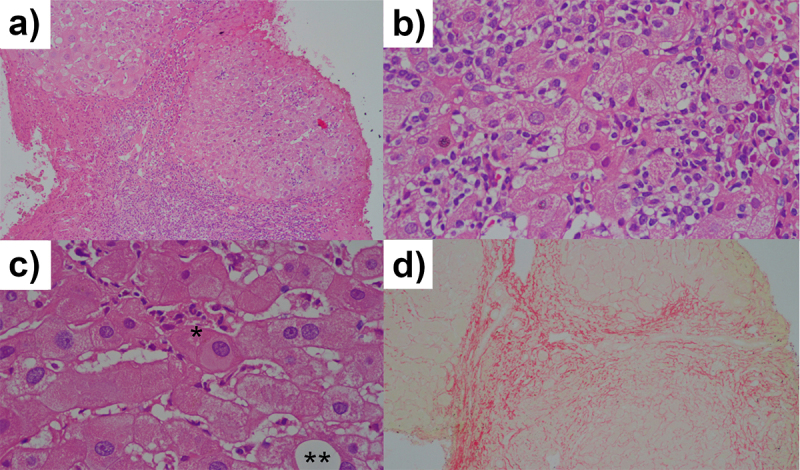
Histopathological sections of liver tissue specimens by hematoxylin-eosin (H&E) staining showed moderate necroinflammatory activity and severe liver fibrosis, corresponding to METAVIR score A2 - F3. **(A)** Liver tissue with fibrous beams outlining regenerating nodules. The fibrous septa are permeated by mixed inflammatory infiltrate. H&E, 200x; **(B)** The infiltration comprises lymphocytes, plasma cells, and eosinophils. H&E, 400x; **(C)** In some fields, HBsAg-containing cells (*) and rare steatotic cells (**) were found. H&E, 400x; **(D)** Marking of collagenous tissue showed the outline of regenerating nodules throughout the sample. Picrosirius, 40x.

The study had a population of 40 patients with chronic infection by HDV, who were positive by serology and with undetectable viral load for HBV, 67.5% (n = 27) with chronic liver disease, evidenced by histopathological analysis illustrated in Figure 1. And 32.5% (n = 13) with no signs of liver parenchyma alteration. Of this total, 70% (n = 28) were male, aged between 23 and 75 years (average of 50.4 years; standard deviation (SD) ± 10.39). For the activity of drinking alcoholic beverages, 32.5% (n = 13) individuals reported doing the practice (Table 2).

**Table 2.** Analysis of the relationship between demographic aspects and the development of CLD in the study population.

Variables	With CLD n (%)	Without CLD n (%)	OR (95% CI)	p-value
Gender				
Male	20 (50%)	8 (20%)	1,786 (0.433 to 6.475)	0.476
Female	7 (17.5%)	5 (12.5%)	0,56 (0.154 to 2.310)	
Age range				
20 - 40 years	6 (15%)	2 (5%)	1.571 (0.289 to 8.576)	> 0.99
41 - 60 years	17 (42.5%)	7 (17.5%)	1.457 (0.424 to 5.176)	0.733
> 60 years	4 (10%)	4 (10%)	0.3043 (0.073 to 1.308)	0.195
Alcoholism				
Yes	10 (25%)	3 (7.5%)	1.961 (0.416 to 7.761)	0.4841
No	17 (42.5%)	10 (25%)	0.51 (0.129 to 2.402)	

The correlation of variables between the groups showed that the development of CLD was not related to age (p > 0.05) or gender (p = 0.476), although CLD patients were mostly male. Alcoholism, another study variable, also showed no association with the development of CLD in the study population (p = 0.4841) (data shown in Table 2).

**Figure 2.**
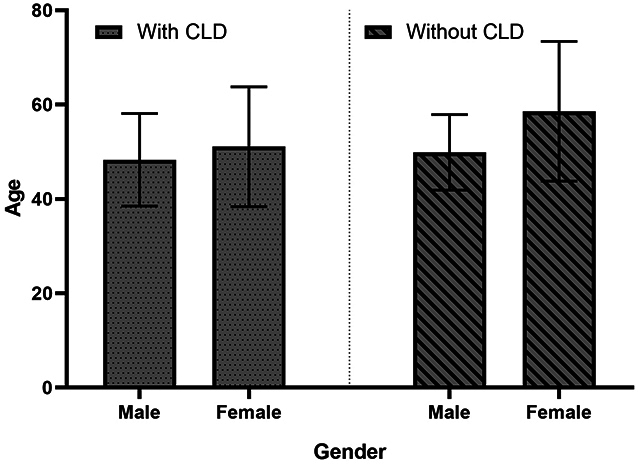
Male individuals with CLD had a mean age of 48.3 (SD 9.8), while non-carriers were 49.9 (SD = 8). Female individuals with CLD had a mean age of 51.1 years (SD = 12.7) and non-carriers 58.6 (SD = 14.8). There was no statistical correlation between gender vs. age vs. CLD (p > 0.05). (p-value = 0.9231 and 0.4206 for male and female gender, respectively).

The correlation of more than two variables, considering the relationship between gender and age with the development of CLD, showed: (1) male subjects with CLD had a mean age of 48.3 years (SD ± 9.8), while subjects without CLD were 49.9 years (SD ± 8); (2) in female subjects, those with CLD had a mean age of 51.1 years (SD ± 12.7) and non-carriers 58.6 years (SD ± 14.8). In both groups, gender and age groups were not associated with the development of CLD (p > 0.05) (Figure 2).

When particularizing the analysis of the influence of gender and age on the development of CLD in individuals who reported alcohol consumption, no significant differences were observed between the groups (p > 0.05). Male alcoholics with CLD were on average 51 years old (SD ± 7.9), and non-carriers were 46 years old (SD ± 5.7). Among female alcoholics, CLD carriers had a mean age of 48 years (SD = 12.7), and non-carriers of 51 years (n = 1) (Table 3).

**Table 3.** Analysis of the relationship between gender and age in alcoholic individuals with the development of CLD in the study population.

Gender	With CLD		Without CLD		p-value
	n (%)	Mean Age (MA)	n (%)	Mean Age (MA)	
Male	8 (61.5%)	51 (± 7.9)	2 (15.3%)	46 (± 5.7)	0.7183
Female	2 (15.3%)	48 (± 12.7)	1 (7.6%)	51 (n = 1)	0.95

### Genetic profile of rs8099917 and rs1800795 in the study population

The frequency of the genotypes and alleles of the SNPs found in the population is shown in Table 4. For the rs8099917 SNP, the TT genotype was the most prevalent, detected in 67.5% of individuals, followed by TG (27.5%) and GG (5%). Consequently, the rs8099917 T allele was the most prevalent relative to the rs8099917 G allele (81.25% vs. 18.75%). All genotypes and alleles were found in higher proportion in individuals with CLD, however, there was no association between the clinical stage of liver disease and the genotype profile of this SNP (p > 0.05) (Table 4).

**Table 4.** Analysis of the relationship of genetic profiles with CLD development in the study population.

Gene (SNP)		With CLD	Without CLD	OR (95% CI)	p-value
		n (%)	n (%)		
IFNL3 (rs8099917)	Genotypes				
	TT	20 (50%)	7 (17.5%)	2.449 (0.693 to 8.894)	0.2836
	TG	5 (12.5%)	6 (15%)	0.2652 (0.056 to 1.048)	0.1278
	GG	2 (5%)	-	-	> 0.999
	Alleles				
	T	45 (56.25%)	21 (25%)	1.429 (0.420 to 4.754)	0.5565
	G	9 (11.25%)	6 (7.5%)	0.7 (0.210 to 2.38)	
IL-6 (rs1800795)	Genotypes				
	GG	18 (45%)	7 (17.5%)	1.714 (0.494 to 5.938)	0.4983
	GC	6 (15%)	5 (12.5%)	0.4571 (0.115 to 1.964)	0.4507
	CC	3 (7.5%)	1 (2.5%)	1.5 (0.202 to 20.93)	> 0.999
	Alleles				
	G	42 (52.5%)	19 (23.75%)	1.289 (0.473 to 3.57)	0.7799
	C	12 (15%)	7 (8.75%)	0.7755 (0.2801 to 2.115)	

For SNP rs1800795, the most prevalent genotype was GG, detected in 62.5% of patients, followed by GC (27.5%) and CC (10%). Thus, the rs1800795 G allele was more prevalent than the rs1800795 C allele (76.25% vs. 23.75%). All alleles and genotypes were observed in higher proportion in individuals with CLD; but, as with the rs8099917 SNP, there was no association between clinical stage and the genotype profile of this SNP in the study population (p > 0.05) (Table 4). Simultaneously, we analyzed the genotype profiles using the Hardy-Weinberg Equilibrium between the groups with CLD and without CLD for the genetic targets, finding genetic equilibrium in the study population (Chi-square < 3.84 = p > 0.05). 

Based on the results obtained in the analyses of CLD development vs. demographic factors and CLD development vs. genetic factors, association analyses were performed among the various variables combined to verify their co-activity in determining the development of CLD in patients with chronic HDV infection in the study population.

The first level of variable matching analyzed was between the different genotypes of the SNPs studied. The highest frequency of combinations was observed among the GG/TT (42.5%), GG/TG (17.5%), and GC/TT (17.5%) genotypes (referring to SNPs rs1800795 and rs8099917, respectively). None of the combinations of genotypes and alleles showed a statistically significant relationship with the development of CLD in the study population, ruling out the joint action of both SNPs with the evolution of liver disease in the cohort studied (Table 5).

**Table 5.** Analysis of the correlation of combinations of the genetic profiles with the development of CLD in the study population

IL-6 rs1800795	IFNL3 rs8099917	With CLD n (%)	Without CLD n (%)	OR (95% CI)	p-value
GG	TT	13 (32.5%)	4 (10%)	2.089 (0.510 to 7.194)	0.337
	TG	4 (10%)	3 (7.5%)	0.580 (0.136 to 2.673)	0.662
	GG	1 (2.5%)	0	-	> 0.999
GC	TT	4 (10%)	3 (7.5%)	0.580 (0.136 to 2.673)	0.662
	TG	1 (2.5%)	2 (5%)	0.211 (0.014 to 2.048)	0.242
	GG	1 (2.5%)	0	-	> 0.999
CC	TT	3 (7.5%)	-	-	0.538
	TG	-	1 (2.5%)	0	0.325

Correlations were then established between (1) CLD development vs. genotypes vs. gender and (2) CLD development vs. genotypes vs. elitism, as described in Table 6. After analysis of the data, it is evident that, regardless of the presence or absence of chronic liver disease, the highest prevalence of genotypes was found in males and that there was no association between combinations of variables (1) and (2). Also, no association with statistical significance was observed in the relationship of genotypes within the variables of gender and alcoholism versus the other genotypes.

**Table 6.** Analysis of the association between genotype and development of CLD with gender and alcoholism in the study population.

Gene (SNP)	Genotypes	Gender				p-value (IC 95%)		
		With CLD		Without CLD				
		Male n (%)	Female n (%)	Male n (%)	Female n (%)	Genotypes *vs.* Gender	Male. *vs*. Other genotypes	Female. vs. Other genotypes
IFNL3 (rs8099917)	TT	14 (35%)	6 (15%)	4 (10%)	3 (7.5%)	0.653	0.4004	0.523
	TG	4 (10%)	1 (2.5%)	4 (10%)	2 (5%)	> 0.99	0.172	0.523
	GG	2 (5%)	0	0	0	-	-	-
IL-6 (rs1800795)	GG	12 (30%)	6 (15%)	4 (10%)	3 (7,5%)	0.673	> 0.99	0.523

**Figure 3.**
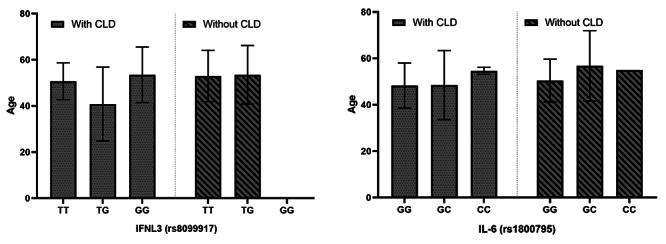
Genotype distribution of rs8099917 (IFNL3) and rs1800795 (IL6) associated with age and the development of chronic liver disease (CLD). Values for p: for SNP rs8099917, genotype TT = 0.8578, genotype TG = 0.1069; SNP rs1800795, genotype GG = 0.9623, genotype GC, 0.5309 and genotype CC > 0.99.

Possible relationships between the genetic profile of SNPs and the age of individuals with the development of CLD were also evaluated, and no relationship was found between these variables (p > 0.05) (Figure 3).

**Figure 4.**
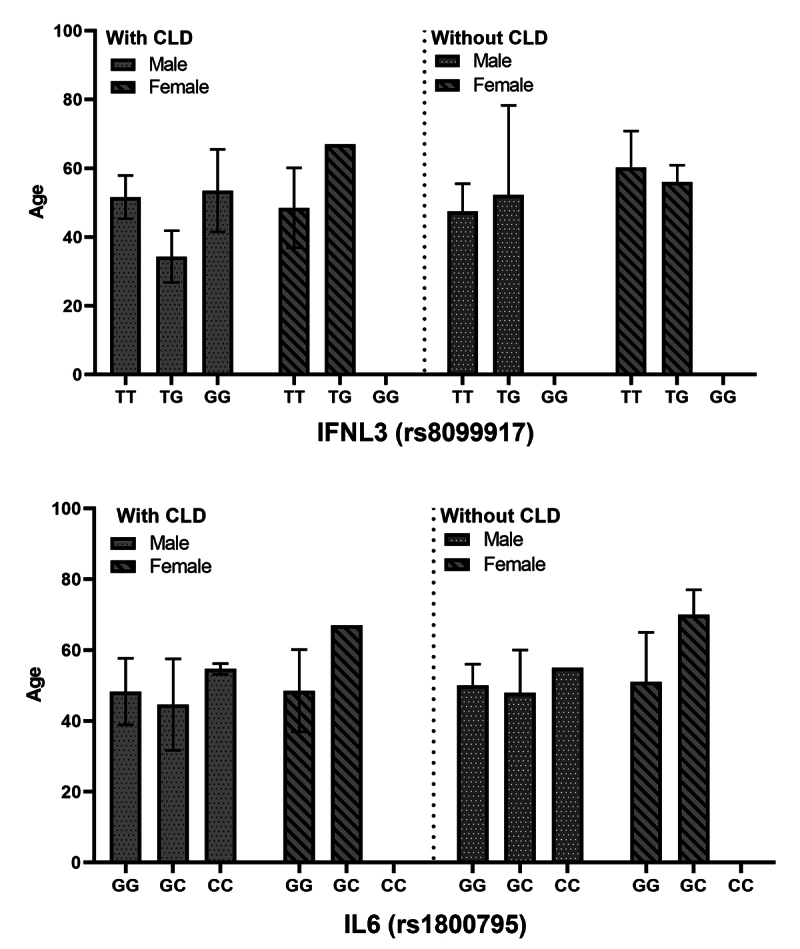
Evaluation of the genotypic distribution of SNPs in relation to the age and gender of the individuals in the study. Values for p: for SNP rs8099917, genotype TT = 0.6221, genotype TG = 0.966; SNP rs1800795, genotype GG = 0.995, genotype GC > 0.99.

Adding the gender of the individuals to this combination, for the association between genotype vs. age vs. gender vs. development of CLD, no significantly relevant relationship (p > 0.05) was also identified, as shown in Figure 4.

**Figure 5.**
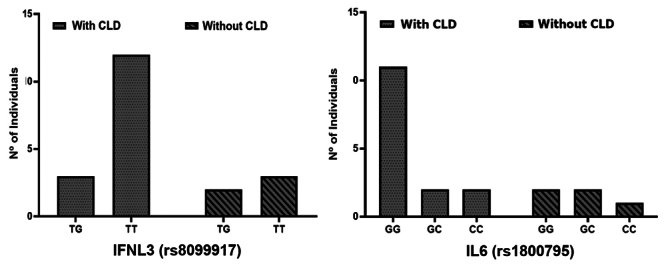
Genotype distribution of rs8099917 (IFNL3) and rs1800795 (IL6) in individuals with thrombocytopenia with and without CLD. Values for p: for SNP rs8099917, genotypes TT = 0.5869 and TG = 0.9876; for SNP rs1800795, genotype GG = 0.5063, GC > 0.999 and CC = 0.9971.

Analyzing the relation of thrombocytopenia and the development of CLD, it was observed that 20 individuals (50%) presented a reduction in platelet count, 15 patients belonging to the group with CLD and 5 to the group without CLD. Thrombocytopenia alone had no statistically significant difference with the development of CLD in the study population (p = 0.5006 and OR = 2 (95% CI between 0.5037 and 7.59). When the genotypic profile of the SNPs of thrombocytopenic individuals was evaluated, with the development of CLD, no statistically significant relationship (p > 0.05) was also found (Figure 5).

**Figure 6.**
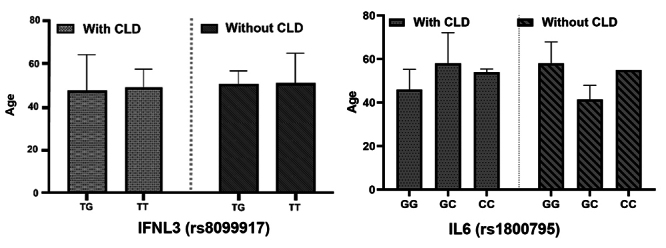
Genotype distribution of rs8099917 (IFNL3) and rs1800795 (IL6) in relation to age in individuals with thrombocytopenia with and without CLD. For SNP rs8099917, among males, the p-value for the TT genotype was 0.2841, and for TG, > 0.99. Among female subjects, the p-value for the TT genotype was 0.6982, and for TG, 0.9785. For SNP rs1800795, among males, the p-value for the GG genotype was 0.1602, GC 0.9740, and CC 0.9740. Among females, the p-value for the GG genotype was 0.6260, CG 0.9740, and CC > 0.999.

Adding age as a factor that could influence this combination, resulting in analysis of genotype vs. age of thrombocytopenic individuals with the development of CLD, no significantly relevant association (p > 0.05) was identified either, as data shown in Figure 6. 

**Figure 7.**
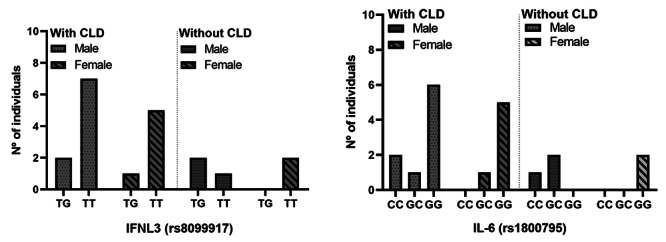
Genotype distribution of rs8099917 (IFNL3) and rs1800795 (IL6) associated with the gender of study subjects with thrombocytopenia. Values for p: for SNP rs8099917, genotypes TT = 0.9489 and TG = 0.9464; for SNP rs1800795, genotype GG = 0.3029, GC = 0.2609 and CC = 0.9997.

The relationship between genotypes of the SNPs of thrombocytopenic individuals vs. gender was also checked, where there was also no association between these variables and the development of CLD in the study population (Figure 7).

## Discussion

The aggressivity of HDV is already well known among specialists, just as it has more developed performances during its natural course of infection [26, 27]. A study in the Amazon region of Brazil analyzed the liver biopsy sample of treated patients with chronic Delta hepatitis, and more than 50% had moderate to severe levels of fibrosis on biopsy [15]. This worsening may be following concerning its pathogenesis which reverberates due to this feature being more striking when seen in coinfections of patients with HBV chronic infection, than when matched to HBV monoinfection [26], which can directly influence the clinical course. 

A diverse range of studies associates genetic polymorphisms concerning several pathologies [28-30], also seeking a way to establish a correlation between the development of the disease and its evolutionary process, since further analysis shows that identifying polymorphisms in cytokine genes and their receptors leads to results that catch the attention of this research, with the high polymorphic variability and the influence in peoples [3]. Most of the polymorphisms identified so far are located in the non-transcriptional regions of the genes, where they can affect expression by inhibiting or stimulating transcription, depending on the regulatory elements and the level of regulation involved [31, 32], which explains the condition of genotypic varieties having supposed favorable or unfavorable conditions even with the treatment/therapy of hepatitis Delta infected persons [33].

Even though the results obtained in this research show that polymorphisms had no direct relationship with the course of HDV infection in patients, the range of SNPs studied for aggravation is immense, emphasizing that work related to pathogenicity and IL-6 gene polymorphisms for HDV has not been performed using rs1800795 so far. This type of mapping within an affected population encourages the use of several targets in different populations, taking into account that rs8099917, of IFNL3, has already addressed its relationship with the treatment of the disease by hepatitis delta, however showing no genetic association with the action of the drug against the virus [34, 35], however, a study has shown that where he demonstrated that in his study population, there was a difference in the genotypes of rs8099917 and rs12979860, belonging to IFNL3, influencing the pegylated interferon (PEG-IFN) response in HDV infection [35].

When analyzing the gender distribution in people infected with HDV [34], it was found that women showed greater representation compared to men, which suggests progressive change facing the viral transmission route. However, divergent results from an analysis performed with IFNL3 polymorphisms have already been seen [34] where men were the majority of those infected with the hepatitis Delta virus. Both studies had no significant results regarding the influence of gender on infection, which corroborates the research results when correlating variables between the groups, showing that the development of CLD was not related to this variable (p > 0.05), although CLD carriers were mostly male.

The age of individuals identified in studies with HDV is varied, and the association of age with the progression of the infection is an approach not so discussed in the literature, however, one study reports that when following patients with acute and chronic Delta virus infection over the long term, when following patients with acute and chronic infection by the Delta virus in the long term, found that there was a less favorable prognosis when in association with the age of the individual at the time of diagnosis [36]. In this study, the relationship made between the age group and the worsening of CLD proved to be absent, which suggests the non-interference of age compared to the progression of the infection.

A possible correlation of IFNL3 and IL-6 gene polymorphisms with the worsening of HDV coinfection was analyzed. One SNP of each research results in 8099917 T/G (IFNL3) and rs1800795 G/C (IL-6), with the remaining variations remaining as promising future targets. The investigation's intent compares it to a study that looked at patients with a progression of infection, which showed the rs8099917 TT genotype related to the severe viral liver infection condition.

The SNP rs8099917 T/G exploration, belonging to IFNL3, showed that the rs8099917 T allele distributed in higher frequency than the rs8099917 G allele in the study population, as previously demonstrated in a study that sought to verify the association of SNP with HBV infection [8, 25]. The mapping performed in a study resembles this one in terms of the distribution of genotypes in the population, however, targeting hepatitis C virus (HCV), of which SNP rs8099917 (IFNL3) was distributed most frequently among TT, followed by TG and GG.

Allele frequency results for the SNP of the IL-6 gene were equally established compared to other studies, with rs1800795 G being the most consistent in both target groups [25, 32]. There was no significant difference between those with CLD and those without CLD, however, according to a study that covered 400 patients and had the principle to study IL-6 polymorphisms in severe cases of HBV infection, their results matched regarding the association of genetic variants at the parasite/host level [37].

In the process of genotypic matching of divergent polymorphisms, analyses of the possible correlation of difference between their most frequent genetic markers were performed, as in a similar study [34, 35]. Both studies showed that when combining polymorphism genotypes, there was no significant difference between those distributed for IFNL3, suggesting that, when combined, they do not influence the parasite/host relationship. Genotype combinations were performed between the IFNL3 genes and, unpublished, with IL-6. In this study, the difference between TT versus GG was significantly absent, as for the other possible combinations.

Another important related variable is thrombocytopenia, present in HBV carriers with liver cirrhosis [38] and already reported in Hepatitis Delta as an aggravating factor in HDV infection [39]. It has already been discussed that the picture of thrombocytopenia in infected individuals, even if seemingly healthy, becomes consistent [38]. Knowing that platelets participate in an important process of hemostasis, one study concluded that in their study with HBV, there is a thrombocytopenic imbalance in cases of liver disease, associating a possible characteristic for individuals with FIB-4 in cases of liver cirrhosis. Although there is no association of thrombocytopenia with the worsening of liver disease for HDV, studies with HBV directed a possible verification in the study population, even if, in these studies, there was no analysis of the genetic relationship between parasite/host. It was verified, from the distribution of the group of individuals with CLD, the frequency of thrombocytopenic being higher than those infected with HDV without liver disease, however, when analyzing this relationship with the genetic profile and demographic factors, the results showed no statistical significance between the variables.

## Conclusion

Even though a diverse range of relationships have been worked out, the results obtained from this study indicate that there is possibly no correlation between the SNPs studied and the worsening of hepatitis Delta virus infection. We conclude that the genotype distribution in the population studied, for the rs8099917 T/G IFNL3 and rs1800795 G/C IL-6 polymorphisms, suggests that there is no relationship to the evolution of chronic liver disease in patients coinfected with hepatitis Delta virus in the northern population of Brazil.

### Abbreviations

IFNL3 - Interferon Lambda 3; IL-6 - Interleukyn-6; CLD - Chronic Liver Disease; HBV - Hepatitis B Virus; HDV - Hepatitis D Virus; SNP - Single Nucleotide Polymorphism; PEG-IFN - Pegylated interferon; HIV - Human Immunodeficiency Virus; HCV - Hepatitis C Virus.
